# Atrial fibrillation‐related stroke in women: Evidence and inequalities in epidemiology, mechanisms, clinical presentation, and management

**DOI:** 10.1002/clc.23284

**Published:** 2019-11-06

**Authors:** Anna Kostopoulou, Hrvojka M. Zeljko, Harilaos Bogossian, Radu Ciudin, Francisco Costa, Jordi Heijman, Simon Kochhaeuser, Sime Manola, Daniel Scherr, Manav Sohal, Reza Wakili, Michael Wolf, Ghazala Irfan, on the behalf of the DAS‐CAM participants—2017‐2018

**Affiliations:** ^1^ Department of Electrophysiology and Pacing Onassis Cardiac Surgery Center Athens Greece; ^2^ Department of Cardiology, Cardiovascular Research Institute Maastricht (CARIM) Maastricht University Medical Center+ Maastricht The Netherlands; ^3^ Faculty of Medicine Josip Juraj Strossmayer University of Osijek Croatia; ^4^ Cardiology Department St Helens and Knowsley Teaching Hospitals NHS Trust, Whiston Hospital Prescot UK; ^5^ Department of Cardiology and Angiology, Klinikum Lüdenscheid Witten/Herdecke University Germany; ^6^ Department of Cardiology “Carol Davila” University of Medicine and Pharmacy Bucharest Romania; ^7^ Department of Cardiology Prof C. C. Iliescu Institute of Cardiovascular Diseases Bucharest Romania; ^8^ Department of Cardiology Hospital Santa Cruz Lisbon Portugal; ^9^ Department of Cardiology II‐Electrophysiology University Hospital of Muenster Muenster Germany; ^10^ Department of Cardiovascular Disease “Sestre Milosrdnice” University Hospital Centre Zagreb Croatia; ^11^ Division of Cardiology, Department of Medicine Medical University of Graz Austria; ^12^ Department of Cardiology, Clinical Academic Group St. George's University Hospitals NHS Foundation Trust UK; ^13^ Department of Cardiology and Angiology Universitätsklinikum Essen, Westdeutsches Herz und Gefäßzentrum Essen Germany; ^14^ Department of Cardiology ZNA Middelheim Antwerp Belgium; ^15^ Department of Cardiac Electrophysiology National Institute of Cardiovascular Diseases Karachi Pakistan

**Keywords:** anticoagulation, atrial fibrillation, hemorrhage, menopause, sex differences, stroke

## Abstract

**Background:**

Atrial fibrillation (AF) is the most common clinical arrhythmia and one of the major causes of stroke, heart failure, sudden death, and cardiovascular morbidity. Despite substantial advances in (interventional) rhythm control treatment during the last decade, anticoagulation for stroke prevention remains a major component of AF treatment.

**Hypothesis:**

There are important sex‐specific differences in AF‐related stroke, resulting from sex‐specific mechanisms and therapeutic differences.

**Methods:**

This review summarizes available data on sex differences in risk assessment and prevention of stroke and highlights current knowledge gaps in AF‐related stroke mechanisms, prevention and management that warrant further research.

**Results:**

Increased thrombotic risk in women is multifactorial, involving hormonal changes after menopause, structural, endocrine and lifestyle/social factors and their interactions. It is clear from randomized studies that women benefit from anticoagulant treatment and that their bleeding risk is similar to men. Women should therefore receive equivalent treatment to men, based on the validated criteria for anticoagulation therapy. However, women are not represented equally in the large randomized studies and sex‐related information in many fields is lacking.

**Conclusions:**

Female sex is an established risk factor for stroke in AF patients. The evidence for sex‐specific differences in stroke risk assessment and stroke prevention is accumulating. However, the underlying biological mechanisms remain incompletely understood and further studies are required in order to decrease AF‐related morbidity and mortality.

## 1 INTRODUCTION

Atrial fibrillation (AF) is the most common clinical arrhythmia and one of the major causes of stroke, heart failure, sudden death, and cardiovascular morbidity worldwide.^1^ The incidence of AF has been rising progressively during the last decade as a result of longer total life expectancy, increased prevalence of comorbidities, lifestyle changes, and improved detection.^1^ The association between stroke and AF increases significantly with age. Approximately 1/3 of total ischemic strokes and more than 50% of those in patients above 80 years of age are associated with AF.^1^ Moreover, patients with AF who suffer an ischemic stroke appear to have a worse outcome, more disability, and greater mortality, than those who have an ischemic stroke in the absence of AF.^2^ Oral anticoagulation (OAC) therapy is an important component of AF treatment, providing significant clinical benefit by preventing ischemic strokes and prolonging life.^1^

## 2 ATRIAL FIBRILLATION IN WOMEN

Although North American and European women at all ages have a lower prevalence of AF compared to men, all‐cause mortality is higher and AF is independently associated with a 2‐fold increase in risk of death in women compared to a 1.5‐fold increase in men.^1^ In the ATRIA study^3^ the annual thromboembolism rate in patients not taking warfarin was 3.5% for women vs 1.8% for men. Females with additional stroke risk factors, particularly older age (>65 years), are at greater risk of stroke, even when adequately anticoagulated, whereas the bleeding risk on anticoagulation was similar in both sexes.^3^, ^4^ Women with AF are typically older, have more comorbidities than men, are more often symptomatic and experience worse quality of life (QOL) and more severe strokes.^3^, ^4^

European guidelines^1^ and a recent review on sex differences in arrhythmias by the European Heart Rhythm Association (EHRA)^5^ underline these sex‐specific issues and recommend offering effective diagnostic tools and therapeutic options to women and men equally. However, in practice, females appear less likely to receive specialist care and when they do, a more conservative approach is followed.^1^, ^6^ The reasons for this are incompletely understood but appear to involve both health provider and patient characteristics. This is a review on sex differences in stroke risk assessment and prevention in patients with AF. It focuses on current knowledge gaps and areas in AF stroke mechanisms and prevention that warrant further research.

## 3 METHODS

Medical literature was reviewed to identify articles investigating atrial fibrillation and stroke risk in terms of pathophysiology and anticoagulation in women. The OVID‐MEDLINE, PubMed, and Cochrane CENTRAL databases were searched for English‐language articles without time restriction. Further selection was based on abstracts and clinical relevance. When available, we focused on randomized controlled trials; if unavailable, important observational studies were used. Our review represents a selection of the main studies published on this topic and is not exhaustive.

## 4 STROKE RISK AND ANTICOAGULATION IN WOMEN

### 4.1 Mechanisms of increased AF and stroke risk in women

**Figure 1:**
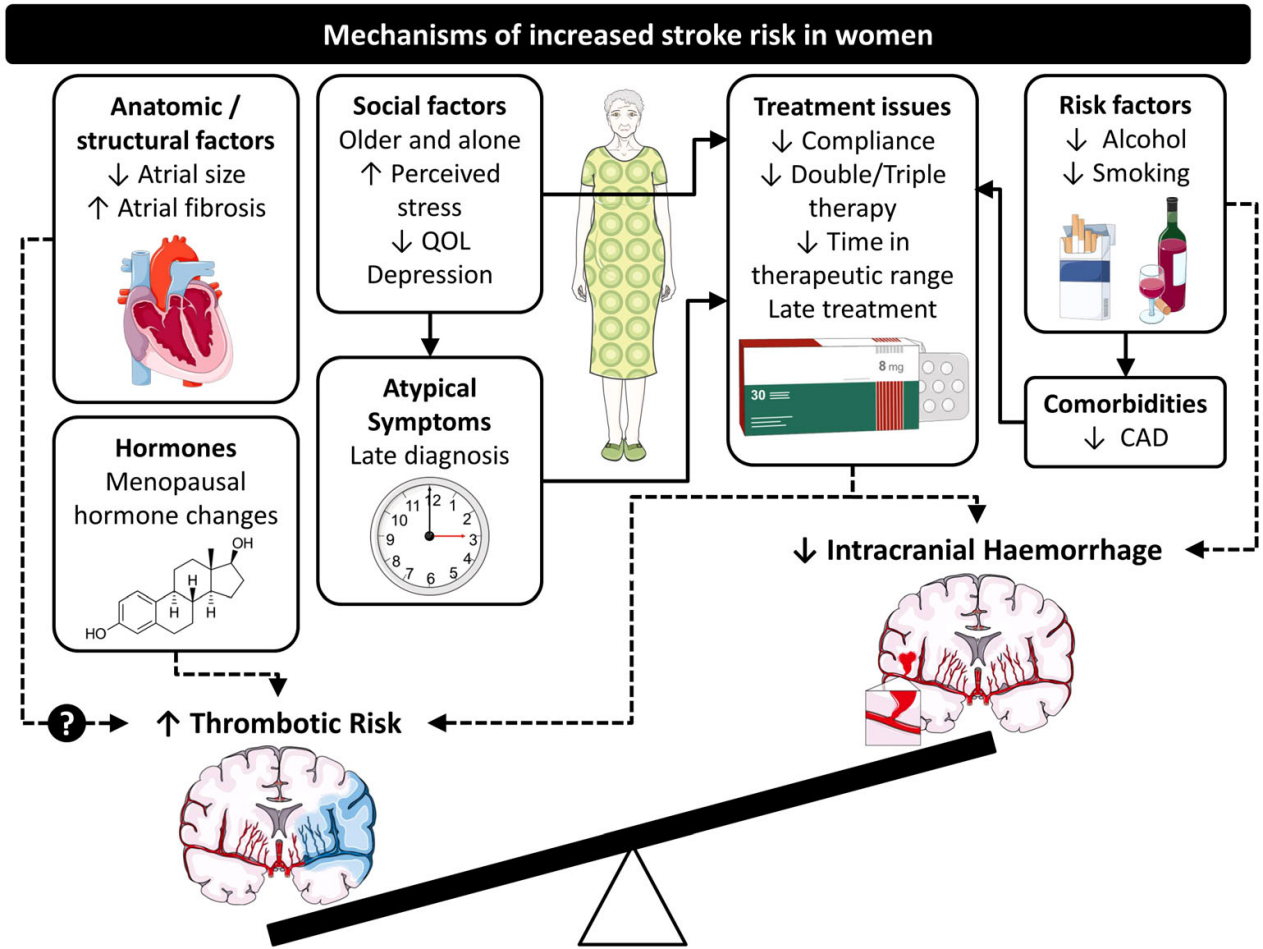
Mechanisms of increased stroke risk in women

The association between female sex and stroke in AF is incompletely understood and a number of clinical and non‐clinical factors have been proposed (Figure 1).

#### 4.1.1 Clinical and cardiometabolic risk factors

Several of the validated clinical predictors of thromboembolism differ between men and women.^3^, ^4^ Women with AF are on average older than male AF patients, in part due to longer life expectancy. In a large retrospective Swedish study^7^ the risk of ischemic stroke was 1.5‐fold higher in women compared to men but this association appeared to be the result of confounding by age. This study showed that in the low risk end, the CHA_2_ DS_2_‐VASc risk score underestimated the ischemic stroke risk conferred by age 65 to 74 years, while it overestimated the risk conferred by female sex.

In addition, women with AF are more often hypertensive but have less coronary artery disease.^3^, ^4^ Although age differences between males and females can be taken into account in the CHA_2_DS_2_‐VASc scheme,^1^ differences in disease severity and quality of treatment, which may also influence stroke risk, are not incorporated, since both hypertension and vascular disease are represented as binary variables.

Renal failure is also a strong predictor of stroke, but is not included in CHA_2_DS_2_‐VASc and no differences between both sexes have been identified.

The association between cardiometabolic factors, AF‐related stroke and sex is incompletely understood. Women with AF seem to be more obese than men but whether this truly increases stroke risk is not clear.^1^, ^8^ A correlation between serum high‐density lipoprotein (HDL), total cholesterol and stroke has been suggested, although these findings are controversial and non‐specific for AF. Surprisingly, in the INTERSTROKE study,^8^ increased HDL was associated with a significantly increased risk of non‐fatal stroke and cerebral infarction in women, whereas men showed an inverse relationship. Similarly, thyroid disorders with increased risk of stroke such as hyperthyroidism are more common in women.^9^ It is however unknown whether females with thyroid disease have a higher thrombotic risk compared to men with thyroid disease. The risk for systemic embolization in the setting of thyrotoxicosis is not precisely known and issues regarding anticoagulation of patients with hyperthyroidism and AF remain unclear.

#### 4.1.2 Endogenous sex‐related hormones

Natural estrogens have many functions apart from maintaining the female reproductive system. Estradiol, the most powerful estrogen has very potent endothelial effects that promote vasodilatation and blood flow through a direct effect on nitric oxide and prostacyclin.^10^ Nitric oxide and prostacyclin, being potent vasorelaxants, inhibit platelet aggregation and adhesion. In the event of an ischemic stroke, estrogens increase energy reserves and decrease reactive oxygen species during reperfusion. Vasoprotection occurs through the preservation of the blood‐brain barrier and the minimization of edema and inflammation.^10^ Estrogens also have direct effects on platelet activity and on the coagulation system through factor VIII and von Willebrand Factor.^11^, ^12^ Menopause leads to a loss of these vasoprotective functions and to increased thrombogenesis as assessed through thromboxane, a product of activated platelets.^11^, ^13^ However, it is not clear whether menopause itself is an independent risk factor for ischemic stroke since higher thrombotic risk has been documented only for women with early menopause.^13^

#### 4.1.3 Hormone replacement therapy (HRT)

In contrast to the protective effect of endogenous female hormones, estrogens prescribed as replacement treatment increase markers of hypercoagulability (eg, factor VIIa) and are known to increase thrombotic risk even in younger groups with additional risk factors.^14^ Earlier studies^14^ showed that HRT was associated with a higher risk of ischemic stroke. However, this has not been confirmed in subsequent studies. In a small group of women on estrogen replacement treatment in the ATRIA study^3^ there was no increase in stroke risk during follow up. Similarly, in a recent sub‐analysis of the AFFIRM study^15^ HRT did not independently predict mortality or thromboembolism. Information and comparisons of different regimens of HRT and stroke risk in women with AF are lacking. Data on the effectiveness and safety of concurrent treatment with HRT and OACs is sparse and conflicting. Some HRT preparations have shown to increase the INR with the risk of acute over‐anticoagulation and hemorrhage.^13^ On the other hand, the AFFIRM trial^15^ did not independently predict mortality, thromboembolism, or bleeding in women on VKAs. When initiating HRT, dose reduction of VKA and close INR monitoring is warranted. Information regarding the safety of NOACs with HRT has not been provided.

#### 4.1.4 Inflammation, oxidative stress, and structural heart disease

A number of structural determinants have been associated with increased stroke risk in AF including left ventricular hypertrophy, left ventricular systolic and diastolic dysfunction, left atrial enlargement, increased appendage size, and valve diseases.^1^, ^16^, ^17^, ^18^, ^19^, ^20^, ^21^ Although women typically have smaller hearts and atria than men, significant changes occur in the presence of AF and AF risk factors.^1^ AF in patients with mitral stenosis or prosthetic valves is classified as “valvular AF” due to the complex pathophysiology and the very high stroke risk.^1^ Although the presence of rheumatic valve disease has decreased, as shown in an epidemiological Swedish registry^17^ three times more women than men have mitral valve stenosis with a 17‐fold increase in the associated stroke risk. The potential sex‐specific impact of other valvular disorders such as mitral regurgitation, aortic or tricuspid valve disease on stroke is unknown. In a study by Cochet et al^19^ delayed‐enhancement magnetic resonance imaging (MRI) of the atria suggested higher levels of fibrosis in women with and without AF. However, the pattern and pathophysiology of fibrosis are not clear and no link to stroke risk has been demonstrated.

There is accumulating evidence about the AF‐promoting role of inflammation and oxidative stress through functional and structural atrial remodeling, for example, from patients with post‐operative AF^20^ or after catheter ablation procedures.^21^ Several anti‐inflammatory therapies have been evaluated, but a systematic decrease in AF burden has not been demonstrated.^21^ However, the underlying molecular mechanisms, as well as the relationship between systemic indices of inflammation and local atrial inflammation remain incompletely understood.^20^, ^21^, ^22^ Systemic CRP levels were independently associated with AF in men, but not in women.^22^ On the other hand, recent data suggest that both female sex and IL‐6 levels are associated with worse survival after stroke.^18^ Accumulating preclinical data also suggest differential microRNA‐mediated regulation of cardiac remodeling as well as responses to cerebral ischemia between males and females, although their role in AF‐related stroke in female patients is still largely unknown.^23^

#### 4.1.5 Autonomic dysfunction

Dysfunction of the autonomic nervous system (ANS) has been implicated in cardiac arrhythmogenesis as well as stroke.^24^ Autonomic triggers can be sympathetic, vagal or mixed. Sympathetic stimulation can trigger AF by promoting ectopic activity. For example, in patients undergoing coronary artery bypass grafting, increased circulating noradrenaline levels and higher resting heart rates due to increased sympathetic tone can induce AF which responds well to peri‐operative beta‐blockade.^20^, ^25^ Vagal AF, on the other hand, refers to a subset of AF patients, who experience episodes in relation to established vagal triggers, including eating, alcohol, nocturnal symptoms or those associated with bradycardia or heart block.^24^ Vagal AF has been suggested to occur primarily in athletes with structurally normal hearts and limited co‐morbidities.^24^ In a Euro Heart Survey substudy, autonomic triggers were found in approximately 1/3 of patients, 41% of them being women.^25^ Surprisingly, vagal AF often occurred in elderly men whose age did not differ from that in the general population and with significant underlying disease. No further sex‐specific analysis was provided in this study, but electrophysiological studies in healthy volunteers have identified male/female differences in the electrophysiological response to activation or blockade of the ANS, with more pronounced shortening of atrial refractoriness in response to ANS activation in women. Additionally, autonomic dysfunction, characterized by altered heart rate variability, was an independent determinant of AF episodes in type 2 diabetic patients^26^ Women and men were equally represented in this study and no sex‐ specific differences were shown. Nonetheless, sex‐specific AF‐promoting mechanisms related to autonomic dysfunction remain an interesting topic for future studies.

#### 4.1.6 Symptoms, life style, and quality of treatment

Women with AF have increased perceived stress, lower QOL, and a higher tendency to develop depression compared to men.^27^ As a result, they more often present with atypical symptoms that can confound the diagnosis and delay treatment. Interestingly, women with AF are more frequently prescribed antidepressants compared to men.^27^ The multi‐center randomized controlled trial SAFETY^28^ showed that women, who were more likely to be older and alone, quite often widows and less educated, have difficulties in seeking medical help. The important issue of under‐treatment of moderate‐to‐high risk patients, in particular women, by physicians has been raised. On the other hand, different studies have shown that although treatment is offered equally, women much more frequently opt for a conservative approach, especially in terms of rhythm management.^6^, ^29^ The AFFIRM trial^30^ and the PINNACLE registry^6^ showed lower quality in OAC treatment in women, suggesting that compliance might be an issue, although this was not confirmed in the ORBIT‐AF registry,^29^ which did not show differences in coagulation rates or times in therapeutic range.

How relevant these issues are in every day practice and whether these differences are due to the healthcare providers, delayed diagnosis, or patients' preferences and compliance represents a real gap in our knowledge.

### 4.2 Sex differences in hemorrhagic risk associated with OAC

Several factors such as age, comorbidities, personal habits (smoking, alcohol), history of bleeding, inappropriate dosing and concomitant use of antiplatelet drugs increase hemorrhagic risk in patients taking OACs.^1^ In a Dutch nationwide study^31^ of drug‐related adverse events, the risk of hospitalization for bleeding related to anticoagulants predominantly vitamin K‐antagonists (VKAs), was higher in men with women having a lower risk of intracranial hemorrhage (adjusted RR 0.51; 95% CI 0.45‐0.58). The ATRIA study^3^ reported a similar rate of major bleeding in both sexes, but again, females had a lower risk of intracranial hemorrhage.^4^ In a pooled analysis^32^ of 25 997 patients on warfarin and 26 944 on non‐vitamin K‐antagonist oral anticoagulants (NOACs) from the 4 major phase III randomized trials (ARISTOTLE, RE‐LY, ROCKET‐AF, and ENGAGE AF^33^) rates of major bleeding on warfarin were comparable for both sexes while women on NOACs had significantly lower bleeding rates compared to men (OR 0.844, 95% CI 0.745 to 0.955, *P* = .007). More information on real‐world data is becoming available from the large global registries currently underway (Table 1).^29^, ^34^, ^35^, ^36^ Among a total of 28 624 patients in the GARFELD‐AF registry,^34^ the risk of major bleeding when treated with anticoagulation vs no anticoagulation was 2.33 (1.41‐3.84) in men and 1.86 (1.16‐2.99) in women (*P* = .53). Overall, these data suggest that the risk of bleeding associated with OAC therapy for AF is broadly similar between both sexes. However, observations suggest that females smoke and drink less, and are less likely to receive double treatment with OAC and antiplatelet drugs, possibly due to less coronary artery disease.^1^, ^3^, ^8^ Moreover, females spend more time below the therapeutic range while on VKAs and this may lead to heightened thromboembolic risk,^6^, ^30^ but could indirectly also contribute to altered bleeding (and/or stroke) risks.

**Table 1:** Global registries on NOACs in patients with AF

	Garfield AF^34^			Orbit AF^29^			Gloria AF^35^			Prefer AF^36^		
	Total	F	M	Total	F	M	Total	F	M	Total	F	M
**Patients**, n (%)	28 624	12 709 (44.4)	15 915 (55.6)	10 135	4293 (42)	5842 (58)	15 092	6872 (45.5)	8220 (54.5)	7243	2546 (35.1)	3866 (64.8)
**Age mean** years ± SD or median IQR		72 ± 10.4	67.6 ± 11.7		77 (69‐83)	73 (65‐80)*		73 (66‐80)	70 (62‐77)		74.1 ± 9.7	70.1 ± 10.7
**CHA**_**2**_ **DS**_**2**_‐**VASC**		4.0 ± 1.4	2.6 ± 1.5		5 (4–6)	3 (2–5)*		4 (3–5)	3 (2–4)		95.2 > 1	78.9 > 1
**HASBLED**		1.5 ± 0.9	1.4 ± 0.9		NR	NR		1 (1–2)	1 (1–2)		70.8 > 1	65.4 > 1
**FU**	1 year			2 years			N/A			1 year		
**Treatment**												
Overall OAC, n (% in total F or M)		NR (63.8)	NR (62.9)		3265 (76.1)	4451 (76.2)	12 065 (79.9)	5474 (79.9)	6591 (80.2)		NR (94)	NR (94)
VKA %		37.1	33.8		71.2	71.2		32.8	31.9		63	61
NOACs %		13.9	13.1		4.9	5.0		46.8	48.3		13	13
VKA + AP %		9.7	12.2								4	7
NOACs + AP %		3.2	3.8									
OAC % (unspecified) + AP					21.5	30.9						
Aspirin %								11.3	11.3			
Unspecified AP %		23.9	24.9									
Other %								0.9	0.9			
None %		12.3	12.2					8.1	7.6			
**All cause mortality**	HR 1.05 (0.92‐1.19)			HR 0.57 (0.49‐0.67)			NR			NR		
**Stroke/SE**	HR 1.30 (1.04‐1.63)			HR 1.39 (1.05‐1.84)			NR			OR 1.08 (0.76‐1.53)		
**Bleeding**	HR 1.13 (0.85‐1.50)			HR 1.03 (0.88‐1.20)			NR			OR 0.91 (0.67‐1.24)		

Abbreviations: AF, atrial fibrillation; AP, antiplatelet; CI, confidence interval; F/M, female/male; HR, hazard ratio; NOAC, non‐vitamin K oral anticoagulants; NR, not reported; OAC, oral anticoagulants; OR, odds ratio; SE, systemic embolism; VKA, vitamin K antagonist.

* *P* < .001 between F&M.

### 4.3 Sex specific differences in anticoagulation therapies

#### 4.3.1 VKAs

VKAs are still the most commonly used medication for stroke prevention in AF due to familiarity with the treatment and their relatively low cost. Early data from the 1990s indicated a higher efficacy of VKA‐treatment in women and greater reduction of thromboembolic risk.^2^ Nevertheless, in a meta‐analysis of the AFFIRM study^30^ a significantly higher residual risk for thromboembolic events was found in women on warfarin (OR 1.279; 95% CI: 1.11‐1.473; *P* = .001). Warfarin use was similar for women and men within their randomization arm over the course of the study. Women had 80 ischemic strokes (5% overall incidence) and men had 77 ischemic strokes (3% overall incidence, odds ratio 1.6, 95% confidence interval 1.19 to 2.26, *P* = .002). Increased events were partially related to the fact that women spent more time outside the therapeutic international normalized ratio (INR) range (40 ± 0.7% vs 37 ± 0.5%, *P* = .0001) and even more importantly, they were more often sub‐therapeutic than men (29 ± 0.7% vs 26 ± 0.5%; *P* = .0002). Moreover, when patients with a fairly high time in therapeutic range (≥66%) were compared,^29^, ^30^ as in the ORBIT study^29^ women still had a higher residual risk of stroke (log rank *P* = .009) and female sex remained an independent risk factor.

#### 4.3.2 NOACs

Landmark NOAC studies^32^, ^33^ did not primarily focus on sex differences and women were underrepresented (37% of 42 411 patients in total, Table 2) in all 4 studies. Nevertheless, initial experience from these studies shows that NOACs have similar safety and efficacy for both sexes. Both rivaroxaban and dabigatran compared with warfarin were associated with reduced risk of heart failure admissions and all‐cause mortality in both sexes. The ARISTOTLE trial showed similar rates of thromboembolism in the apixaban and warfarin groups between men and women but less clinically relevant bleeding in women. Although firm data is lacking and studies with direct comparisons of NOACs in women and men are not available, the existing information indicates that NOACs can be used equally in women and men.

**Table 2:** Risk of major bleeding in women compared with men with AF treated with NOACs and warfarin

Study name	Total No. Women %	Bleed/total treated with NOAC			Bleed/total treated with warfarin		
		Women	Men	*P* value	Women	Men	*P* value
ARISTOTLE	35.46%	102/3234	225/5886	.101	168/3182	294/5899	.541
RE‐LY 150 mg	35.75%	141/2150	268/3865	.579	148/2236	273/3840	.468
ROCKET‐AF	39.64%	135/2819	260/4292	.023	133/2826	253/4299	.032
ENGAGE AF‐TIMI 48^33^	38%	Total number of bleeds not mentioned			Total number of bleeds not mentioned		

Abbreviations: AF, atrial fibrillation; NOAC, non‐Vitamin K oral anticoagulants.

### 4.4 Anticoagulation around interventions

#### 4.4.1 Cardioversion and ablation

Controlled data and evidence on sex‐related outcomes with regard to cardioversion and ablation are scarce and sex differences are rather absent. There are few randomized controlled trials (RCTs) on cardioversion and women are underrepresented in most (Table 3).^37^, ^38^, ^39^, ^40^, ^41^, ^42^, ^43^, ^44^, ^45^, ^46^ The events in these trials were very low and no comparisons between men and women could be made. The most recent EMANATE trial^39^ enrolled women equally distributed, to evaluate the use of apixaban compared to VKA/heparin with respect to outcomes of thromboembolism, stroke, and bleeding. Due to the very low event rate in the trial, no real sex‐specific differences could be detected. Nevertheless, OAC by NOACs or VKA/heparin seem to be equally safe and effective in women undergoing direct current cardioversion.

**Table 3:** Studies with anticoagulation around interventions

Study name (cardioversion studies)	Total No. women %	Total treated with NOAC		Total treated with VKA/Heparin		Study results
		Women	Men	Women	Men	
**X‐Vert** 37 (Cardioversion‐Rivaroxaban vs VKA)	27.5%	275/1002	727/1002	135/502	367/502	Low event rate. No comparison between genders made
**ENSURE‐AF** 38 (Cardioversion‐Endoxaban vs VKA)	34%	374/1095	721/1095	382/1104	722/1104	No gender specific differences in outcome
**EMANATE** 39 (Cardioversion‐ Apixaban vs VKA/Heparin)	30.8%‐38.7%	248/753	505/753	250/747	497/747	Low event rate. No gender‐related differences were detected
**Study name (RF ablation studies)**	**Total No. women %**	**Total treated with interrupted VKA**		**Total treated with interrupted VKA**		**Study results**
		**Women**	**Men**	**Women**	**Men**	
**COMPARE** 40 (RF Ablation‐ interrupted vs uninterrupted Warfarin)	25%	188/790	602/790	204/794	590/794	Sub‐group analysis did not show any gender specific differences
		**Total treated with uninterrupted NOAC**		**Total treated with uninterrupted VKA**		
**VENTURE AF** 41 (RF Ablation‐ Uninterrupted Rivaroxaban vs VKA/heparin)	29%	38/124	86/124	34/124	90/124	Small study Low event rates. Both gender benefit on uninterrupted NOAC
**RE‐CIRCUIT** 42 (RF ablation‐uninterrupted Dabigatran vs VKA/heparin)	25%	87/317	230/317	73/318	245/318	Both gender benefit on uninterrupted NOAC
**Study name (PCI Studies)**		**Total treated with NOAC**		**Total treated with VKA/Placebo**		**Study results**
		**Women**	**Men**	**Women**	**Men**	
**RE‐DUAL‐PCI** 43	22%‐25%	424/1744	1320/1744	401/1745	1344/1745	No gender differences in terms of safety and efficiency
**PIONEER AF‐PCI** 44 (Rivaroxaban with DAPT for 1‐month, 6‐months, and 12‐months)	24%‐26%	543/2124	1581/2124	N/A	N/A	No gender differences in terms of safety and efficiency
**WOEST** 45 (OAC with Clopidogrel or Clopidogrel plus Aspirin after PCI)	17%‐23%	N/A	N/A	N/A	N/A	No gender differences in terms of safety and efficiency
**ATLAS ACS II‐TIMI 51** 46 (Rivaroxaban vs placebo in ACS)	25%	2632/10 350	7718/10 350	12 194/5176	3882/5176	No gender differences in terms of safety and efficiency

Abbreviations: ACS, acute coronary syndrome; DAPT, double antiplatelet treatment; N/A, non‐applicable; NOAC, Non‐vitamin K oral anticoagulants; PCI, percutaneous coronary intervention; RF, radiofrequency; VKA, vitamin K antagonist.

Uninterrupted OACs during ablation were first studied in the COMPARE trial^40^ which showed significant benefits for the continued OAC arm with respect to stroke, TIA, and bleeding. While 25% of the study cohort was female, the subgroup analysis failed to show any sex specific differences. The VENTURE‐AF^41^ and RE‐CIRCUIT studies^42^ tested uninterrupted rivaroxaban and dabigatran, respectively, vs VKA/heparin periprocedurally. The VENTURE‐AF‐trial was a very small trial with very low events rates whereas in RE‐CIRCUIT there was a consistent benefit of uninterrupted NOACs vs uninterrupted VKAs with respect to bleeding events in both females and males, although bleeding rates were numerically higher in both arms.

#### 4.4.2 Acute coronary syndromes—Percutaneous interventions and major surgery

Although women are underrepresented in randomized clinical trials, they are believed to be more susceptible to cardiovascular events with higher mortality and increased bleeding complications after PCI and full antiplatelet treatment (Yentl syndrome). However, in the four major studies, RE‐DUAL‐PCI,^43^ PIONEER AF‐PCI,^44^ WOEST,^45^ and ATLAS ACS II‐TIMI 51 trial,^46^ in which 20% to 25% of patients were female, no sex differences were identified in terms of safety and efficiency of dual or triple therapy (Table 3) and indications for double and triple treatment are identical for both men and women. As for major surgery there are no female‐specific studies or any special OAC recommendations.

### 4.5 Special situations

#### 4.5.1 Pregnancy

AF is very unusual in young pregnant females with structurally normal hearts, but may occur in pregnant patients suffering from heart failure, cardiomyopathies or congenital heart disease.^1^ Anticoagulation should be guided by the available risk assessment tools, though these are not validated in pregnancy. When VKAs are the indicated anticoagulant, although low dose warfarin was shown to be safe in pregnant women, higher doses may have adverse effects on fetal outcome as they can cross the placenta and are therefore discontinued after the first trimester.^1^ Information on potential consequences of NOACs during pregnancy is sparse. Currently, only one observational study is available assessing risks in women inadvertently exposed to rivaroxaban during early pregnancy, but the small cohort does not allow to refute an increased malformation risk. Beyer‐Westendorf et al,^47^ recently reviewed 233 unique cases of NOACs in pregnancy. Outcome was available in 137 (58.8%) cases. Seven (5.1%) showed abnormalities from which 3 (2.2%) could be potentially interpreted as embryopathy. The authors concluded that data was incomplete and women exposed to NOACs should therefore not be directly advised to terminate pregnancy but change to LWMH.

#### 4.5.2 Chronic kidney disease

Limited data are available for OACs in patients with renal disease and sex‐specific information is lacking.

## 5 OUTCOME AFTER STROKE

Women with AF present more frequently with stroke compared to men and have worse outcome due to multiple factors and characteristics, including smaller body mass index and older age.^1^ Intracranial hemorrhage is the most feared complication of OAC treatment and is associated with high mortality and morbidity. In patients with previous intracranial hemorrhage, restarting OAC treatment as early as clinically feasible, was associated with significantly lower risk of ischemic stroke and all‐cause mortality, without statistically significant risk of recurrent bleeding.^1^ Unfortunately, these patients are usually not included in RCTs, and the problem of the best time window for restarting the OAC after intracranial hemorrhage, taking into account potential sex differences, remains an issue of debate.

## 6 LIMITATIONS

This review includes multiple studies of non‐homogenous design and representation of females (randomized non randomized and observational). Females are underrepresented in most studies and drawing conclusions incorporates a bias risk. In addition, a major limitation of current studies is the fact that most of them were conducted in Europe or North America. To what extend sex‐specific aspects are relevant for diagnosis and treatment in other parts of the world is at present largely unknown.

## 7 CONCLUSION

Female sex is an established risk factor for stroke in AF patients. The evidence for sex‐specific differences in stroke risk assessment and stroke prevention, especially OAC treatment is accumulating. However, the underlying biological mechanisms remain incompletely understood and there are many gaps in evidence that need to be addressed. Advancing the knowledge in this field will likely help to decrease AF‐related morbidity and mortality and to further personalize treatment.

## CONFLICT OF INTEREST

The authors declare no potential conflict of interests.
